# Supplementary material for: Relationship between Pelvic Dimensions and Maximum Traction Forces Required during Parturition in Holstein Cows Using a Biomechanical Obstetric Simulator
Source: Animals (Basel). 2024 Jul 8;14(13):2011. doi: 10.3390/ani14132011 (PMC11240830; doi:10.3390/ani14132011)
Supplement: Supplementary file 1 [file animals-14-02011-s001.zip › animals-3088744-supplementary.pdf]

SUPPLEMENTARY MATERIAL

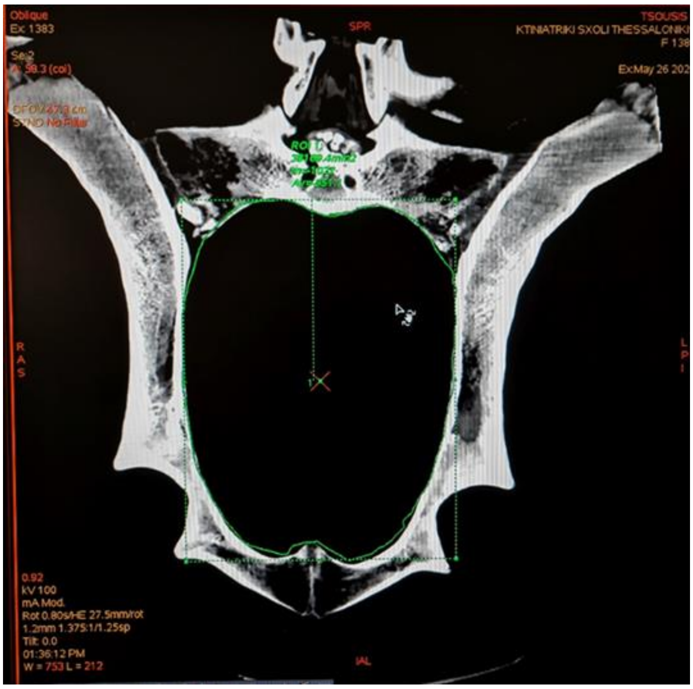

Figure S1: Pelvic area (PA) as it was measured with CT scan.

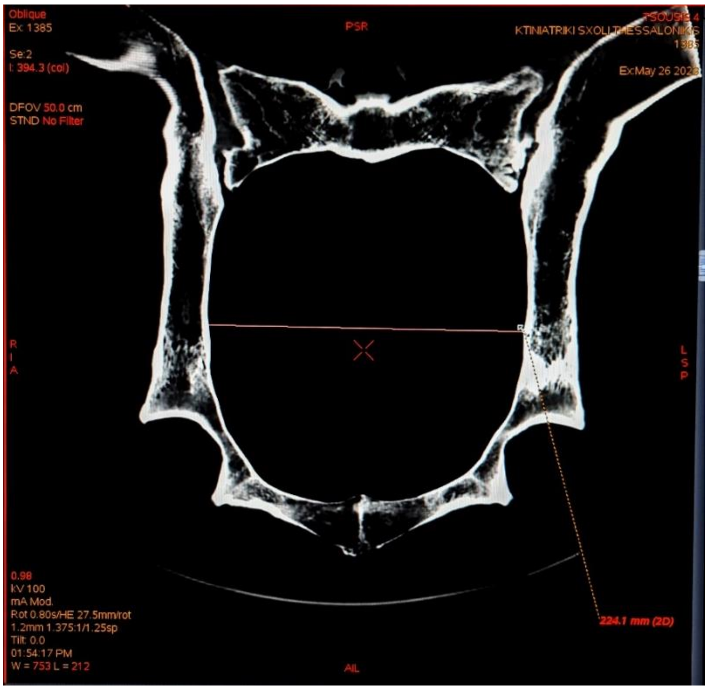

Figure S2: Medial horizontal diameter of pelvic inlet (Dhpi) as it was measured with CT scan.

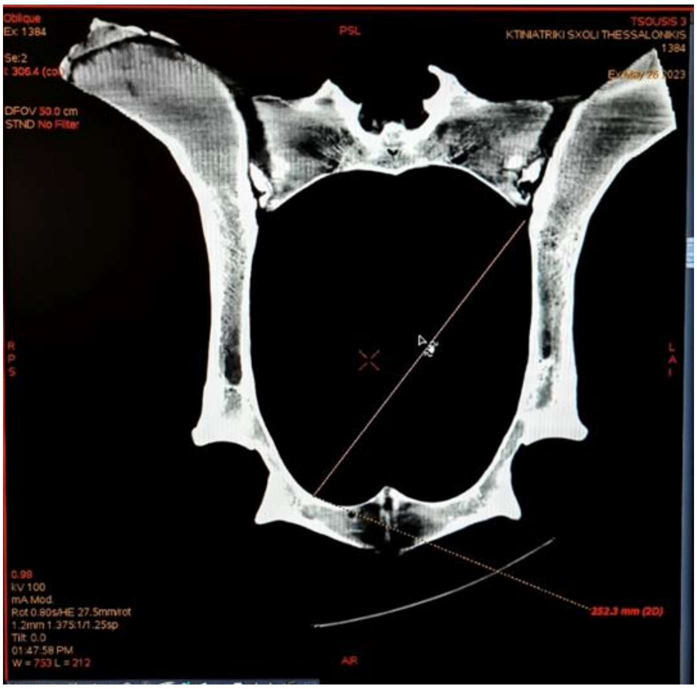

Figure S3: Diagonal diameter of pelvic inlet (Dia) as it was measured with CT scan.

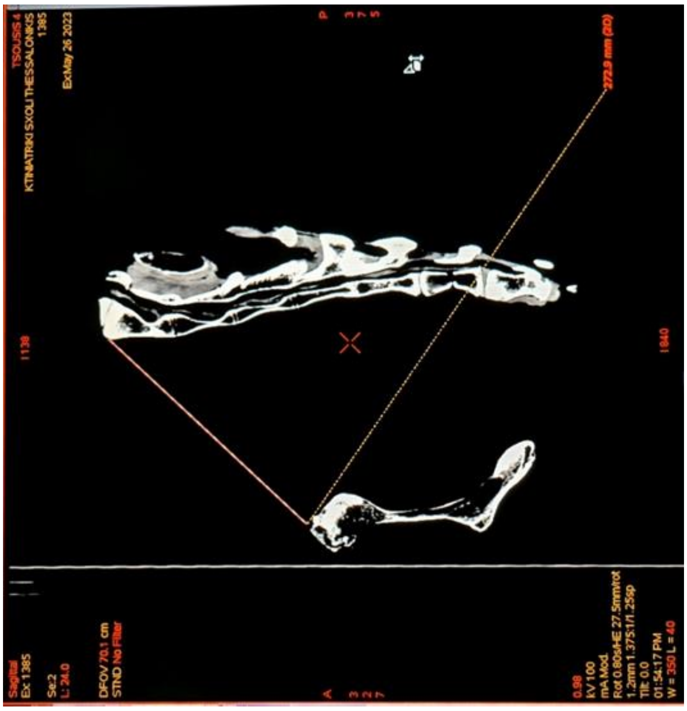

Figure S4: Height of pelvic inlet (Hpi)as it was measured with CT scan.

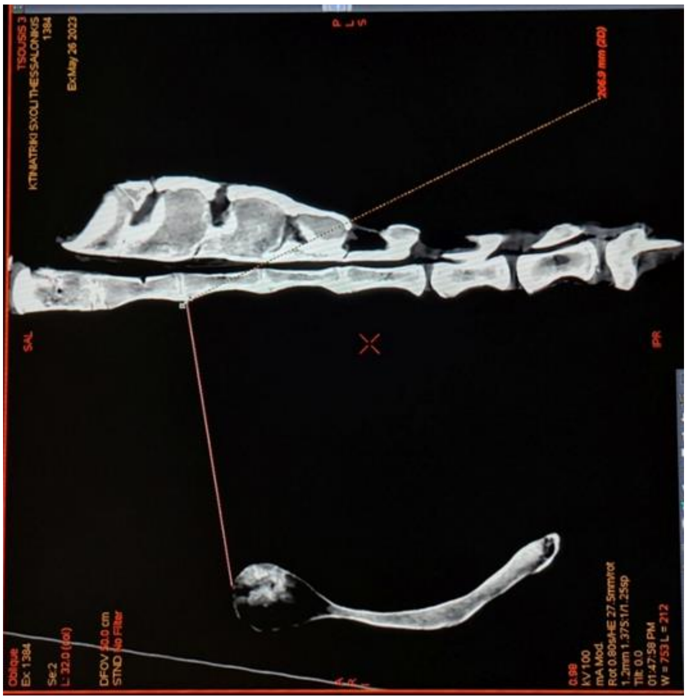

Figure S5: Minimum height of pelvic cavity (Hmin) as it was measured with CT scan.

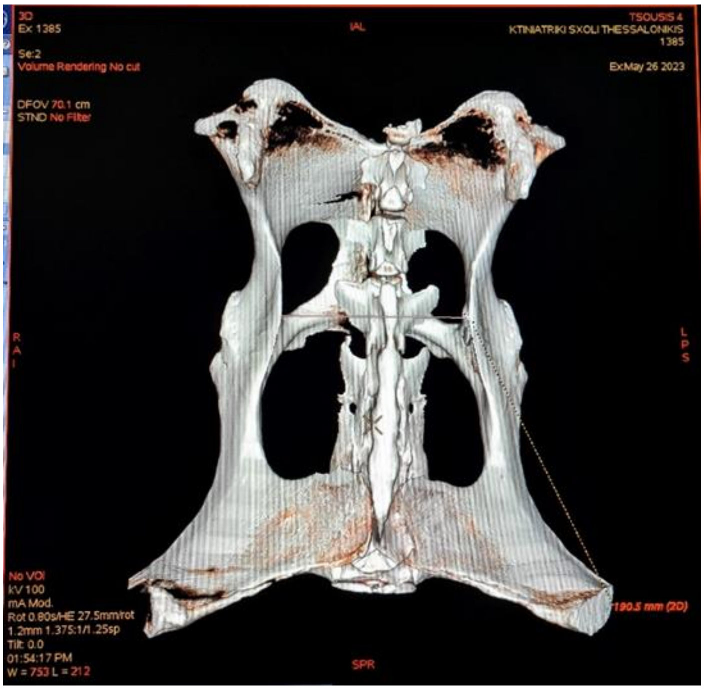

Figure S6: Narrowest horizontal diameter of midpelvis (Dhmin) as it was measured with CT scan.

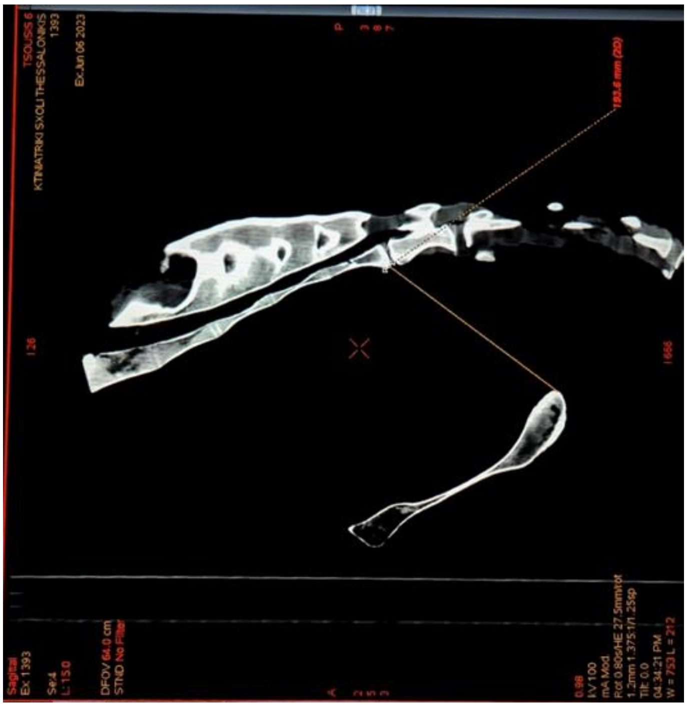

Figure S7: Caudal height of pelvic cavity (Hc) as it was measured with CT scan.

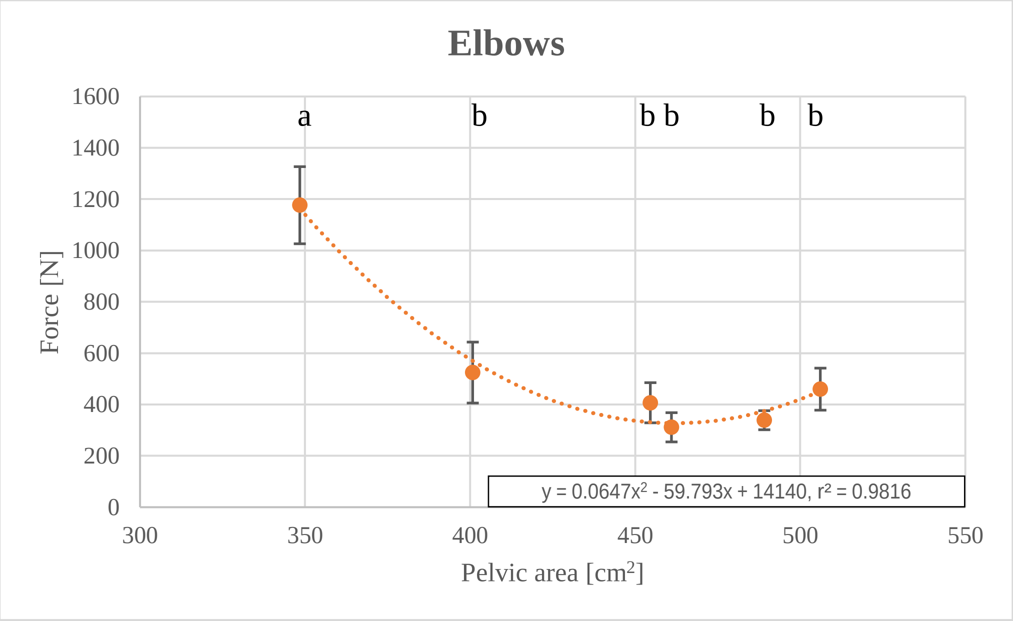

Figure S8. Maximun traction forces recorded during the entrance of the elbows in the pelvic cavity in association with pelvic inlet area. [a,b] : Different exponents indicate statistically significant difference between two consecutive measurements.

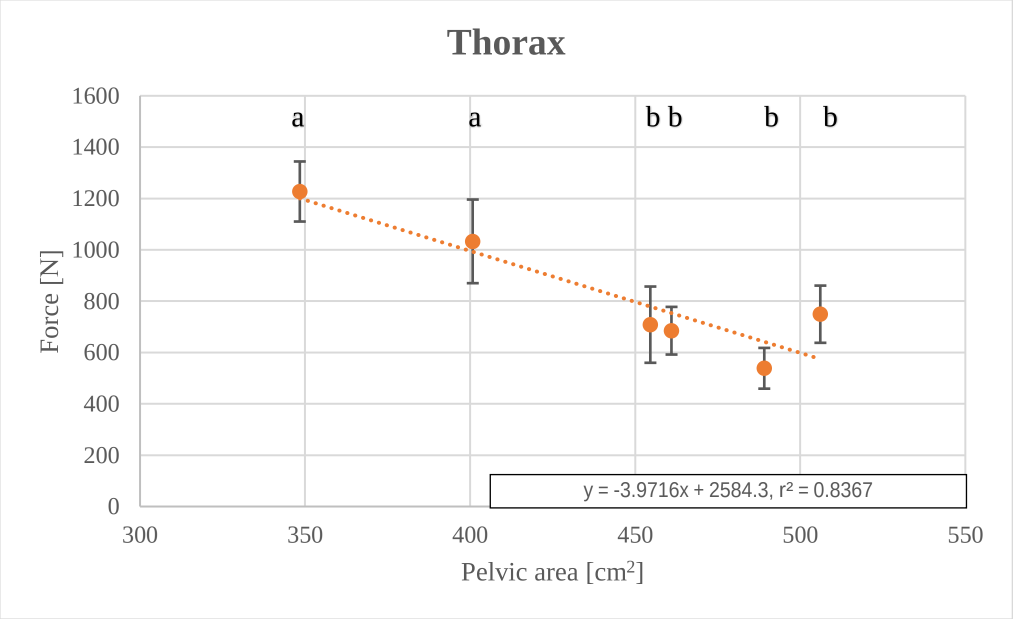

Figure S9. Maximun traction forces recorded during the entrance of the thorax in the pelvic cavity in association with pelvic inlet area. [a,b] : Different exponents indicate statistically significant difference between two consecutive measurements.

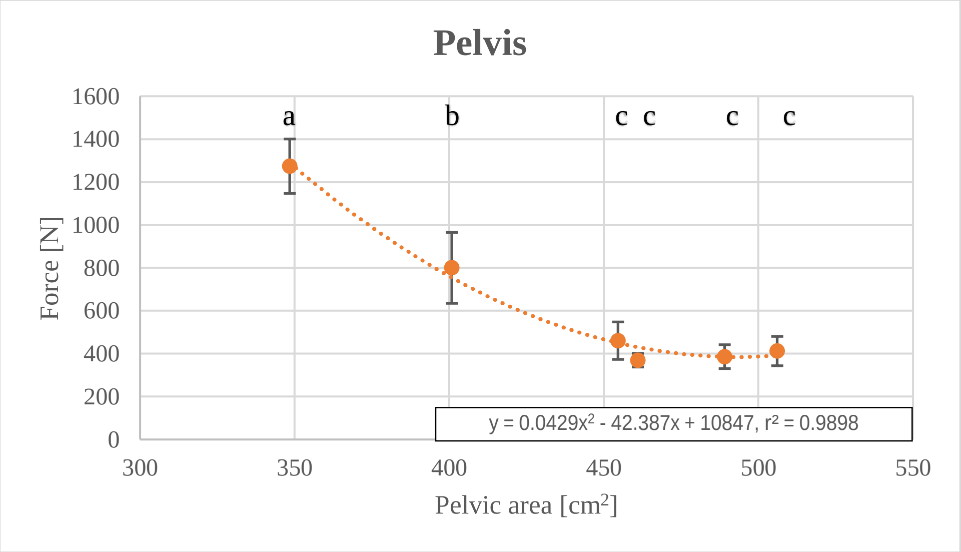

Figure S10. Maximun traction forces recorded during the entrance of the calf's pelvis in the maternal pelvic cavity in association with pelvic inlet area. [a,b] : Different exponents indicate statistically significant difference between two consecutive measurements.

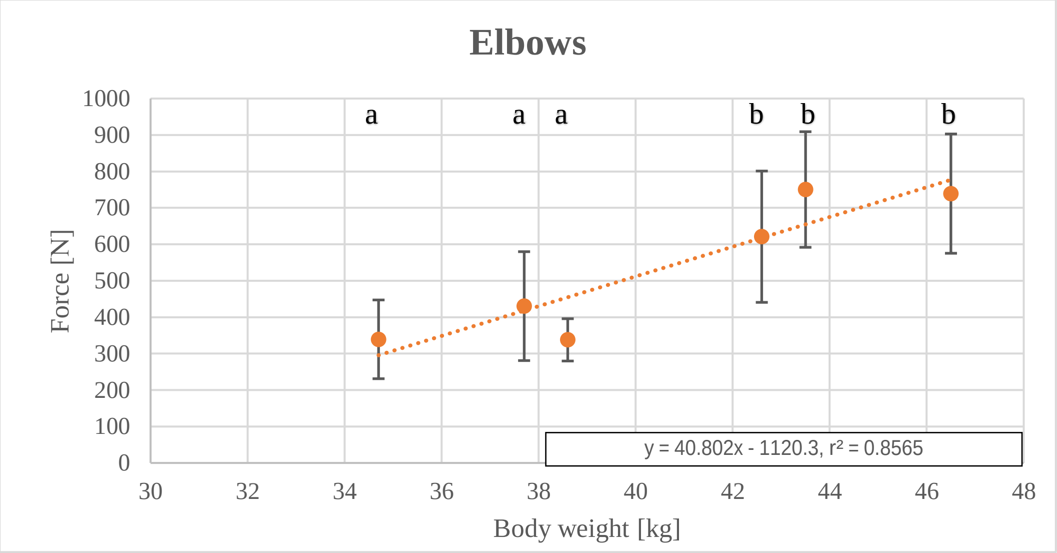

Figure S11. Maximun traction forces recorded during the entrance of the elbows in the maternal pelvic cavity in association with calf body weight. [a,b] : Different exponents indicate statistically significant difference between two consecutive measurements.

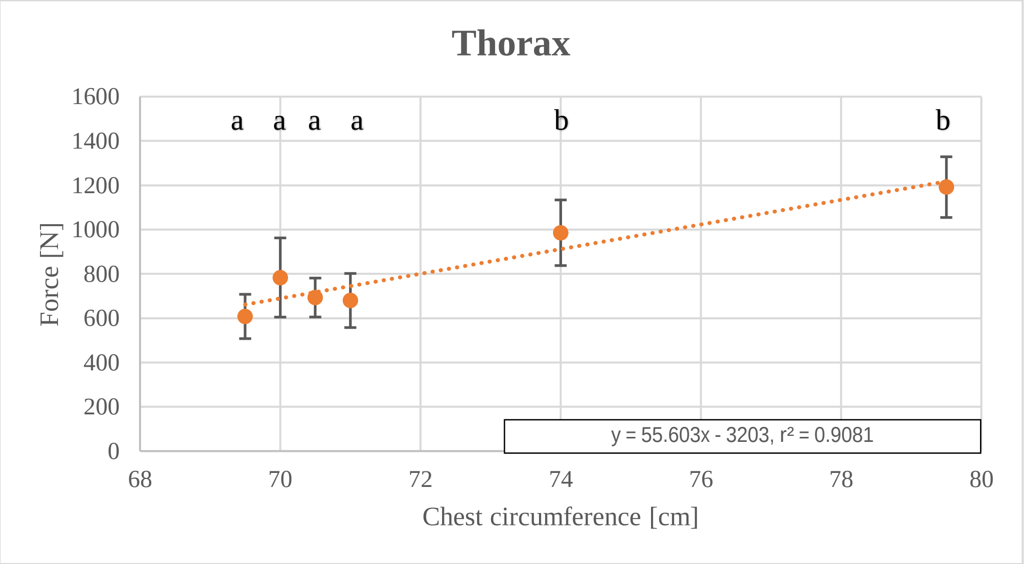

Figure S12. Maximun traction forces recorded during the entrance of the thorax in the maternal pelvic cavity in association with calf chest circumference. [a,b] : Different exponents indicate statistically significant difference between two consecutive measurements.

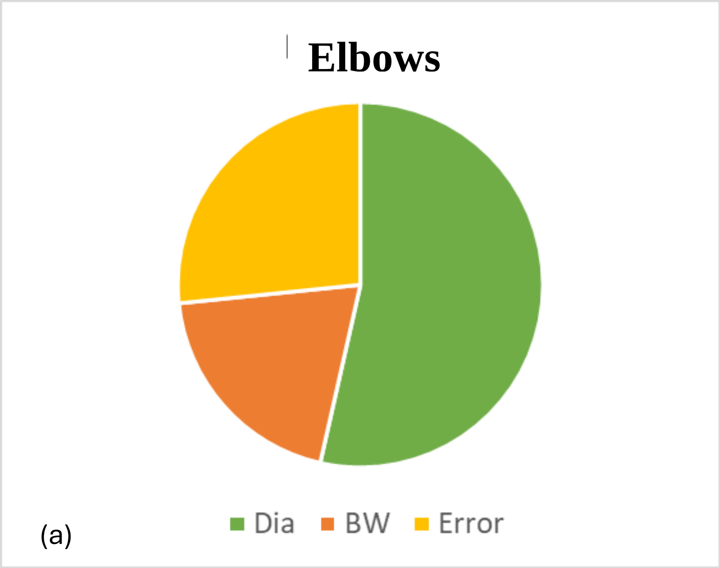
Elbows
Dia
BW
Error
(a)

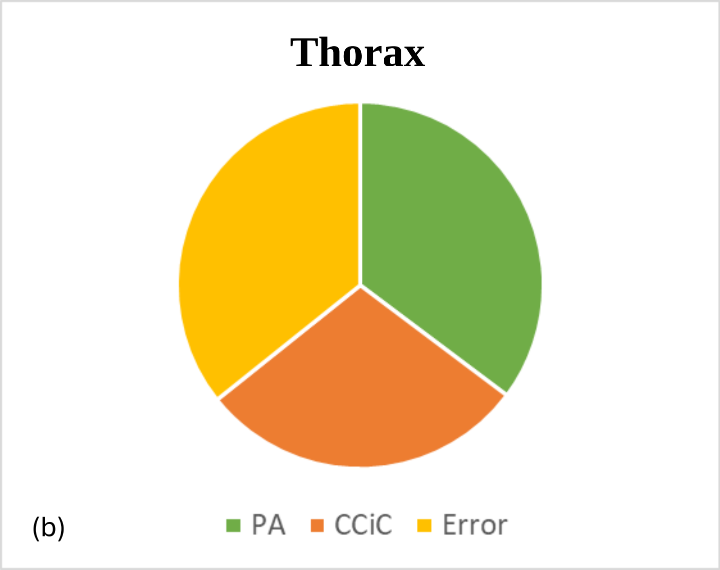
Thorax
PA
CCiC
Error
(b)

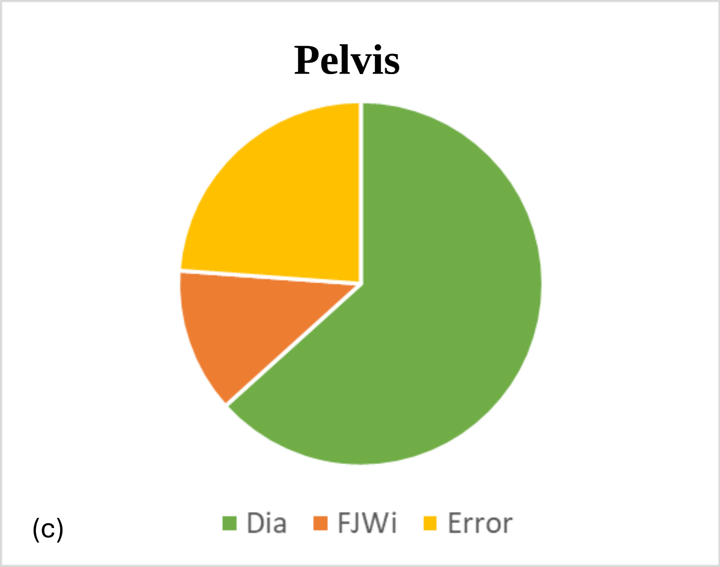
Pelvis
Dia
FJWi
Error
(c)

Figure S13. Contribution of pelvic (green) and calf (orange) parameters on the variance of traction forces during the entrance of the elbows (a), thorax (b) and pelvis (c) using sum of square type 3.

Dia: Diagonal diameter of pelvic inlet, BW: Body weight (Calf), PA: Pelvic inlet area, CCiC: Chest circumference (Calf), FJWi: Fetlock joint width (Calf)

Table S1. Results of multivariate regression analysis containing all variables below 0.10 for the entrance of the elbows (ELBOWS), the entrance of the chest (CHEST) and the entrance of the pelvis (PELVIS).

Body Entrance Area	Variable	Estimate	SE	t Value	Pr > |t|	Adj- r^2
ELBOWS						0.83
	intercept	5322	1533	3.47	0.0016	
	Dia	-402	64	-6.26	<.0001	
	Hpi	204	50	4.08	0.0003	
	PA	17	3.8	4.50	<.0001	
	Hmin	-459	126	-3.65	0.0010	
	BW calf	41	6.4	6.39	<.0001	
CHEST						0.69
	intercept	-8269	2733	-3.03	0.0051	
	Dhpi	112	64	1.74	0.0922	
	PA	-15	4.8	-3.20	0.0032	
	Hmin	464	179	2.60	0.0144	
	BW calf	17	9.6	1.72	0.0961	
	CC calf	47	11.0	4.31	0.0002	
PELVIS						0.75
	intercept	5873	580	10.12	<.0001	
	Dia	-136	15	-9.36	<.0001	
	FLJW	-373	88	-4.21	0.0002	

SE: Standard error; Dia: Diagonal diameter of pelvic inlet; Hpi: Height of pelvic inlet; PA: Pelvic area; Hmin: Minimum height of pelvic cavity; BW: Body weight; Dhpi: Medial horizontal diameter of pelvic inlet; CC: Chest circumference; FLJW: Fetlock joint width;
